# Has new rural pension system reduced the intake of junk food among rural older adults? Evidence from China

**DOI:** 10.3389/fpubh.2023.1131337

**Published:** 2023-03-13

**Authors:** Shi Purun, Zhengxiu Sun, Jiaying Cao, Zhile Li

**Affiliations:** ^1^School of Business, Nanjing Audit University, Nanjing, China; ^2^School of Economics and Management, Southeast University, Nanjing, China; ^3^School of Government, Nanjing University, Nanjing, China

**Keywords:** new rural pension system, dietary health, junk food, income shock, fuzzy regression discontinuity

## Abstract

At present, China has become one of the fastest growing countries in terms of junk food consumption. However, there has been less previous evidence for the effect of endowment insurance on dietary health. Using the data China Family Panel Studies (CFPS) from 2014, this paper exploits a policy, the New Rural Pension System (NRPS), that only the older adults who have reached 60 years old can receive pensions and conduct a fuzzy regression discontinuity (FRD) to address endogeneity and examine the causal effect of the NRPS on the intake of junk food among rural older adults in China. We find that the NRPS can significantly reduce junk food intake among them, which remains robust after a series of robustness tests. In addition, heterogeneity analysis shows that the female, low-educated, unemployed, and low-income groups are more sensitive to the pension shock from the NRPS. The result of our study provides insights to effectively improve people's dietary quality and related policy formulation.

## 1. Introduction

Diet has an important impact on human health, economic development, and social labor productivity (1–3). In 2017, 11 million deaths and 255 million disability-adjusted life year (DALY) worldwide are attributed to unhealthy dietary factors (4). Notably, unhealthy diet has become the 5th most important risk factor for disease and death (5). As for China, some studies have shown that the dietary quality of Chinese residents is not high and there are some problems with the nutritional structure (6, 7). The intake of junk food is likely to be an important inducement. According to statistics, China's dietary quality is deteriorating due to unhealthy pattern and China has become the one of the fastest growing countries in terms of unhealthy items consumption (8).

Junk food is generally high in salt, sugar and fat (9) and has become the main source of food for people at the bottom of society due to its relatively low price (10). However, numerous studies have shown that such unhealthy diet is directly harmful to health (11, 12). Specifically, the intake of high sugar increases cardiometabolic risk (13, 14). The high intake of sodium and preserved vegetables predisposes to hypertension (15, 16). Typical “Western dietary patterns,” including refined cereals, red meat and its products, sugars, pastries, and fried potato chips, etc., significantly increase cardiovascular morbidity and mortality (17). The intake of excessive fat increases obesity rates (18, 19), which leads to a variety of cancers (20). Therefore, it is worthwhile explore how to reduce unhealthy food to improve health status.

Income is likely to be an important factor in changing the dietary health and nutritional structure (13, 14, 21, 22). Specifically, sustained income growth led to higher food expenditures (23). Yu and Abler (24) found that rural households in China purchased more high-quality food as their income increases. Li et al. (25) found income has a significant contribution to the dietary health of rural residents. Huang and Zhang (26) found the pension from the NRPS improves the nutrition intake, which is in line with the study of the effects of the social pension in Korea (27). However, negative income shocks led to a significant reduction in household intake of calories, fat, and protein (28). Income risk might cause individuals to decrease their meat consumption (29). In addition, the main diets of low-, middle-, and high- income households were fruits and dairy, meat, and cereals, respectively (30). In fact, other factors, such as age, education, marriage, gender, place of residence, occupation, household size, caloric intake of animal foods, etc. (21, 30–35), play important roles in people's diet as well. In summary, there are few studies that focused on the effect of income on the intake of junk food.

However, there are a few challenges to accurately identify the above causal relationship. The main one is the endogeneity caused by the relationship between income and diet. The above seems to prove that income has an influence on diet. However, individual nutritional intake and levels due to diet may influence income as well (36–39). Therefore, China's New Rural Pension System (NRPS) provide us with a valuable natural experiment, an exogenous income shock. It stipulates that those who have reached 60 years old can receive pensions, which is designed to provide basic and stable livelihood security for the older adults aged above 60.

This paper contributes to the strand of literature that examines the effects of the NRPS. Most of extant studies focused more on its effect on labor supply, child transfer payments, medical service utilization, mental health, and health inequality (38, 40–43). However, we innovatively complement the effect of the NRPS from the perspective of dietary health. In addition, we further corroborate the role of income in improving diet structure and provide institutional-level support. Our implications may provide useful information to policymakers in China, even in other developing countries, on the reform of the future social security system and how to improve residents' dietary quality.

The remainder of the paper is organized as follows. Section 2 briefly introduces China's NRPS. Section 3 describes the data, descriptive statistics, and empirical strategy. Section 4 presents the main results and tests for identification validity. Section 5 presents heterogeneity analysis. Section 6 is discussion. Section 7 concludes.

## 2. China's NRPS

The New Rural Pension System (NRPS) is a large social pension insurance system launched by the Chinese government for the purpose of guaranteeing the basic livelihood of the elderly in rural regions. In September 2009, the General Office of the State Council of the People's Republic of China officially issued the “Guidance Opinions of the State Council on Launching the Pilot Program of the New Rural Pension System,” marking the beginning of the pilot program of the NRPS. According to incomplete statistics, in the same year, the first 320 pilot counties were identified, and the number of participants reached 72.77 million. In 2010, the number of pilot counties was further expanded to 838, and 30 million new participants were added. In 2011, the number of pilot counties was further expanded to 1,914, and 220 million new participants were added. By the end of 2012, China's NRPS has covered the whole country, with 460 million people insured (44, 45). Notably, the NRPS is an unprecedented welfare program covering the largest population in human history (26).

In terms of the participation, the NRPS stipulates that the principle of voluntary participation in the household registration was implemented for those rural residents who have reached the age of 16 and have not participated in the Urban Employees' Basic Endowment Insurances (UEBEI),^1^ excluding students and soldiers. In fact, this combination of government-led and voluntary system has greatly expanded the coverage of the NRPS.

In terms of the funding model, it is a combination of individual payments, collective subsidies, and government subsidies. Specifically, enrollees can choose their own payment level which is adjusted in accordance with relevant indicator such as the net per capita income of rural resident. The more you pay, the more you get. In addition, some village collectives give subsidies to enrollees, and the government encourages organizations or individuals other than village collectives to provide financial support for NRPS accounts. Some local governments subsidize each enrollee by no < 30 yuan per year and provide an additional subsidy to those who participate in high-level security.

In terms of the pension management and distribution, the social pooling account and the individual account are set up. Monthly pension benefits comprise the basic pension from social pooling account and the individual pension from the individual account. Specifically, the social pooling account is fully financed by government funds. The basic pension from it is not < 55 yuan per month. Individual payment and subsidies from collective and government are credited to individual accounts. The government is responsible for making investment decisions and managing the funds in individual accounts. The monthly individual pension is 1/139 of the total accumulation in the individual account.

## 3. Data, descriptive statistics, and empirical strategy

### 3.1. Source of data

The data used in this paper derives from the China Family Panel Studies (CFPS), a collaborative effort between the China Social Science Survey Center at Peking University and the University of Michigan Survey Research Center, U.S.A. The CFPS is designed to track data at the individual, household, and community levels. It is a nationwide, large-scale, multidisciplinary social tracking survey covering a wide range of variables including economic activity, educational outcomes, family relationships and dynamics, population migration, and health. Since FRD is locally randomized, no tracking data are required. Eventually the 2014 cross-sectional data are selected for our analysis in this paper. Given that we focus on the effect of the NRPS on the intake of junk food and that the CFPS only ask questions about the NRPS to residents aged 45 years and older, only the samples of rural hukou aged 45–75 years are retained for this paper,^2^ and the samples with the missing question “The intake of junk food in the past week” are excluded.

7-day dietary food record is widely chosen as the reference method and applied to studies in the field of nutrition (e.g., 46–49). It records the respondents' intake of a certain food in the past 7 days. Similarly, the 2014 CFPS inquires whether meat, fish and other aquatic products, fresh vegetables and fruits, dairy products, soy products, eggs, pickled foods, puffed/ fried foods, and miscellaneous grains were consumed in the past week.^3^ Among them, pickled foods and puffed/fried foods respectively contain high salt and high oil, which are typical representatives of junk foods. Therefore, we set the dependent variable *Junk food intake* as a dummy variable which takes the value 1 if the person has consumed pickled, puffed, or fried food in the past week, and 0 if otherwise.

### 3.2. Descriptive statistics

Table 1 shows the basic information of all the variables used in this paper.

**Table 1:** Summary statistics.

	**Obs**	**Mean**	**p50**	**Std dev**	**Min**	**Max**
Junk food intake	9,995	0.480	0	0.500	0	1
Gender	9,995	0.496	0	0.500	0	1
Education	9,995	1.902	2	0.983	1	5
Self-rated health	9,995	3.307	3	1.280	1	5
Work status	9,995	0.202	0	0.402	0	1
Household incomes per capita	9,995	9,775.647	7,340	9,763.446	0.833	117,665
Age_60.25	9,995	−3.212	−3.583	8.203	−15.500	15.500
Pension	9,995	0.211	0	0.408	0	1

Gender is a dummy variable which takes the value 1 if the person is male, and 0 if otherwise. Education is a continuous variable which equals 1 if the person is illiterate, 2 if the person receives elementary school education, 3 if the person receives junior high school education, 4 if the person receives senior high school or technical secondary school education, and 5 if the person receives junior college education and above. Self-rated health is a continuous variable which equals 1 if the person considers himself/herself rather healthy, 2 if the person considers himself/herself very healthy, 3 if the person considers himself/herself healthy, 4 if the person considers himself/herself a little unhealthy, and 5 if the person considers himself/herself unhealthy. Work status is a dummy variable which takes the value 1 if the person has jobs, and 0 if otherwise. Household incomes per capita is the average income of the person's household excluding pensions. Age_60.25 is a continuous variable after respondents' age is standardized. Pension is a dummy variable which takes the value 1 if the person receives the pension from the NRPS, and 0 if otherwise.

Source: CFPS2014.

Table 2 shows the result of subgroup statistics. In Table 2, we demonstrate the mean on both side of the cutoff. In the selected baseline sample aged 45–75, Column (1) and Column (2) show that the mean of the intake of junk food in the past week is 0.495 on the left side of the cutoff and 0.455 on the right side of the cutoff, respectively. The mean on the right side of the cutoff is significantly lower than that on the left side at the 1% level. Meanwhile, the mean of other variables is also significantly different on both sides of the cutoff. Among them, the left side of the cutoff is significantly better than the right side in terms of education, self-rated health, and work status. The possible reason is that the samples on the left side of the cutoff is younger overall. Therefore, a direct comparison of only the mean on both sides of the cutoff cannot verify the causal effect of the NRPS on the intake of junk food. Moreover, Column (3) and (4) show that the differences in other control variables narrow when the age range is limited to 2 years on both sides of the cutoff. By contrast, the mean of the intake of junk food in the past week is 0.514 on the left side of the cutoff and 0.453 on the right side, with the difference between the mean on both sides being significantly larger and significant at the 5% level, which is possibly the result of the mitigating effect of the NRPS. Only approximate information of the sample can be observed through descriptive analysis. The specific causal effects request more rigorous empirical analysis.

**Table 2:** Subgroup statistics.

**Variables**	**[45–60.25)**	**[60.25–75]**	**Diff**	**[58.25–60.25)**	**[60.25–62.25]**	**Diff**
	**(1)**	**(2)**	**(3)**	**(4)**	**(5)**	**(6)**
Junk food intake	0.495 (0.500)	0.455 (0.498)	−0.040^***^	0.514 (0.500)	0.453 (0.498)	−0.061^**^
Gender	0.487 (0.500)	0.512 (0.500)	0.025^**^	0.472 (0.500)	0.477 (0.500)	0.005
Education	2.111 (1.025)	1.532 (0.776)	−0.579^***^	1.799 (1.028)	1.600 (0.872)	−0.199^***^
Self-rated health	3.168 (1.275)	3.554 (1.252)	0.386^***^	3.481 (1.256)	3.453 (1.287)	−0.028
Work status	0.261 (0.439)	0.099 (0.299)	−0.162^***^	0.165 (0.372)	0.130 (0.337)	−0.035^*^
Household incomes per capita	10,679.450 (10,129.870)	8,177.779 (8,858.006)	−2,501.671^***^	8,980.302 (8,820.643)	9,265.875 (9,036.743)	285.573
Pension	0.018 (0.002)	0.550 (0.008)	0.532^***^	0.062 (0.009)	0.425 (0.018)	0.363^***^
Observation	6,384	3,611		757	777	

The values above parentheses represent the mean values of the variables. The values in Column (3) represent the mean difference between Column (1) and (2), i.e., (2)–(1) = (1). The values in Column (6) represent the mean difference between Column (4) and (5), i.e., (5)–(4) = (6). Analysis in Column (1) includes samples aged 45–60. Analysis in Column (2) includes samples aged 61–75. Analysis in Column (4) includes samples aged 59 and 60. Analysis in Column (5) includes samples aged 61 and 62. Standard deviation in parentheses. ^***^p < 0.01, ^**^p < 0.05, ^*^p < 0.1.

Source: CFPS2014.

### 3.3. Empirical strategy

#### 3.3.1. Fuzzy regression discontinuity

Regression discontinuity (RD), emerging and being widely used in recent decades, plays an important role in identifying causal effects. The basic concept of this identification strategy is to use discontinuous features on the policy rule. This policy rule allows an individual to be treated when an observable characteristic variable, i.e., forcing variable, is equal to or greater than a threshold, i.e., cutoff. As long as the individual is not able to fully manipulate the forcing variable, the discontinuous changes in the dependent variable can be considered to be caused by the treatment state. However, in many cases, although the treatment state is a discontinuous function of the forcing variable, it does not necessarily change from 0 to 1 at the cutoff, but only increases the probability that the treatment state takes the value 1 (52). In this case, the RD we use becomes fuzzy regression discontinuity (FRD). Then, the treatment and control groups are constructed on both sides of this cutoff to obtain the local average treatment effect (LATE) which represents the causal effect (53). In fact, scholars generally believe that FRD is closer to quasi-natural experiments, and the estimated results are more accurate (43).

**Figure 1:**
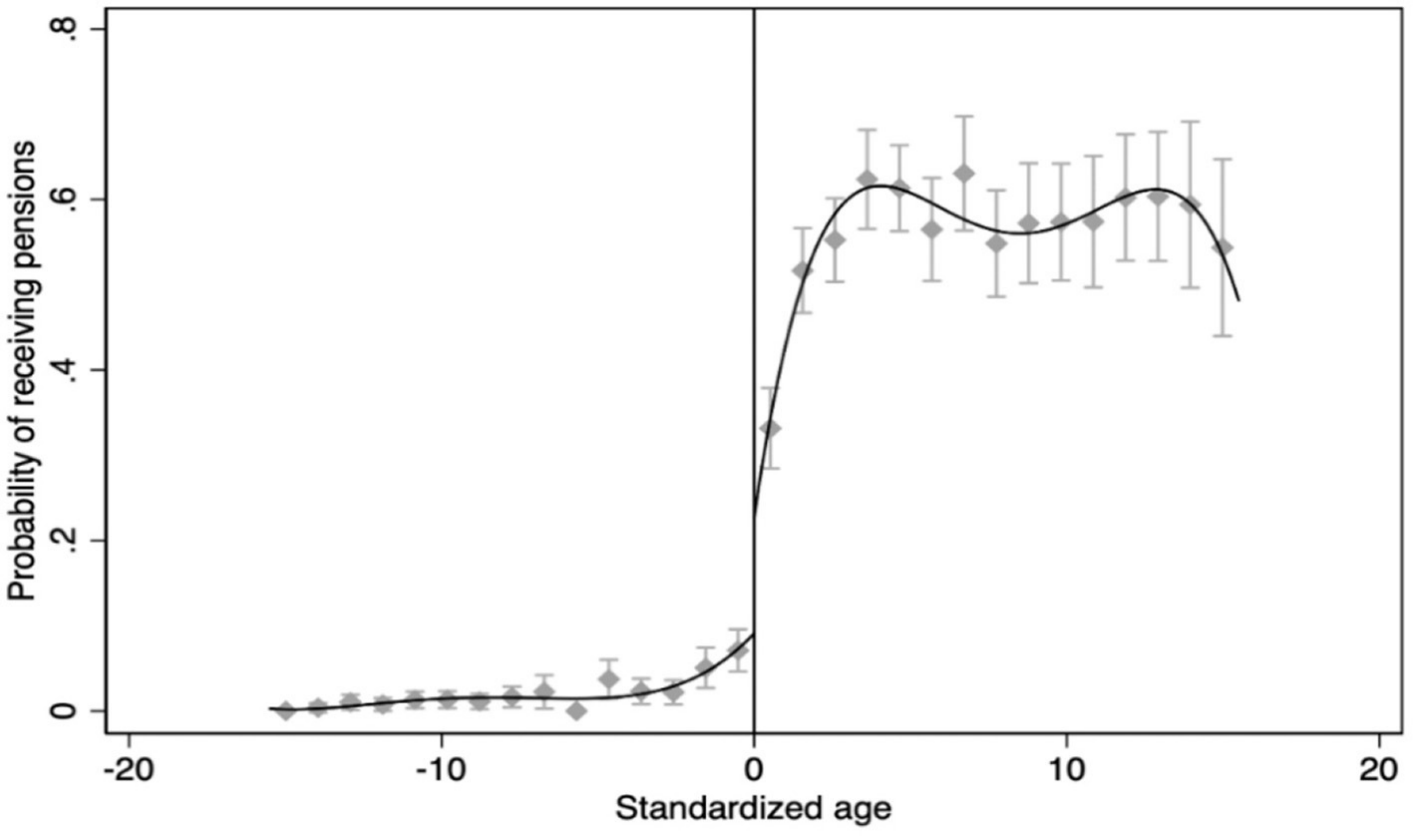
The cutoff in the probability of receiving pensions at the standardized age 0. Source: CFPS2014 and Stata 16.0.

Figure 1 shows the probability of receiving pensions for the rural older adults. It can be observed that there is a significant upward jump in the probability of receiving pensions at the standardized age 0, i.e., age 60. The reason is possibly that, in the practical implementation of the policy, there are phenomena of pensions being paid before or over the age of 60 due to the preference of local governments to pay out in accordance with the actual situation, though the NRPS stipulates that pensions can be received upon reaching the age of 60. Therefore, the possibility of receiving pensions jumps < 1 at the cutoff instead of changing from 0 to 1, which is consistent with the setting of the FRD. Based on the above, we use FRD to examine the causal effect of the NRPS on the intake of junk food among rural older adults in China.^4^

In this paper, we set age as the forcing variable and 60.25 years old as the threshold^5^, i.e., cutoff. According to Calonico et al. (54) and Calonico et al. (55), the estimate τ can be presented by the following equation (1).


(1) $$\begin{array}{l} \tau \;=\;\frac{E\left[Y_{i}\left(1\right)|X\;=\;0\right]-E\left[Y_{i}\left(0\right)|X\;=\;0\right]}{E\left[D_{i}\left(1\right)|X\;=\;0\right]-E\left[D_{i}\left(0\right)|X\;=\;0\right]\;} \end{array}$$

Where *D*_*i*_ equals 1 if pensions are received and 0 if otherwise. *Y*_*i*_ is the dependent variable, i.e., the intake of junk food in the past week. *Y*_*i*_(1) is the potential intake of the junk food assuming that pensions are received, i.e. *D* = 1. *Y*_*i*_(0) is the potential intake of the junk food assuming that pensions are not received, i.e., *D* = 0. *X* is the standardized age.

Due to the probability of receiving pensions jumping on *X*, *E*[*D*_*i*_(1)|*X* = 0] − *E*[*D*_*i*_(0)|*X* = 0] ≠ 0, which makes the consistent estimate of τ as follows.


(2) $$\begin{array}{l} \tau \;=\;\frac{\tau_{Y}}{\tau_{D}}\;=\;\frac{\mu_{Y+}-\mu_{Y-}}{\mu_{D+}-\mu_{D-}} \end{array}$$

In equation (2), $\mu_{Y+}\;=\;\underset{x\to 0^{+}}{\lim}\mu_{Y}\left(x\right)$, $\mu_{Y-}\;=\;\underset{x\to 0^{+}}{\lim}\mu_{Y}\left(x\right)$, $\mu_{D+}\;=\;\underset{x\to 0^{+}}{\lim}\mu_{D}\left(x\right)$, $\mu_{D-}\;=\;\underset{x\to 0^{+}}{\lim}\mu_{D}\left(x\right)$, μ_*Y*_(*x*) = *E*[*Y*_*i*_|*X*_*i*_ = *x*], μ_*D*_(*x*) = *E*[*Y*_*i*_|*X*_*i*_ = *x*], so


(3) $$\begin{array}{l} \tau \;=\;\frac{\tau_{Y}}{\tau_{D}}\;=\;\frac{\underset{x\to 0^{+}}{\lim}E\left[Y_{i}|X_{i}\;=\;x\right]-\underset{x\to 0^{-}}{\lim}E\left[Y_{i}|X_{i}\;=\;x\right]}{\underset{x\to 0^{+}}{\lim}E\left[D_{i}|X_{i}\;=\;x\right]-\underset{x\to 0^{-}}{\lim}E\left[D_{i}|X_{i}\;=\;x\right]} \end{array}$$

Using the minimization method to estimate equation (3), the results are as follows.


(4) $$\begin{array}{l} \tau_{D}\;=\;\arg\underset{\alpha ,\beta ,\tau ,\gamma }{\min}\sum_{i\;=\;1}^{N}1\left\{c-h\leq X_{i}\leq c+h\right\}\\ \;{\left(D_{i}-\alpha -\beta \left(X_{i}-c\right)-\tau D_{i}-\gamma \left(X_{i}-c\right)T_{i}\right)}^{2} \end{array}$$

(5) $$\begin{array}{l} \;\tau_{y}\;=\;\arg\underset{\alpha ,\beta ,\tau ,\gamma }{\min}\overset{N}{\underset{i\;=\;1}{\sum }}1\left\{c-h\leq X_{i}\leq c+h\right\}\\ {\left(Y_{i}-\alpha -\beta \left(X_{i}-c\right)-\tau D_{i}-\gamma \left(X_{i}-c\right)T_{i}\right)}^{2} \end{array}$$

Where *T*_*i*_ is the eligibility for pensions, which equals 1 if *X*_*i*_ ≥ 60.25 and 0 if otherwise. *D* and *T* are highly correlated but not equal under the structure of FRD. *h* is the bandwidth.

In addition, one of the keys in non-parametric estimation is the choice of bandwidth. Therefore, we choose the minimum mean square error method proposed by Calonico et al. (55) to select the optimal bandwidth, which can well balance validity and credibility, i.e., MSE[$\tilde{\tau }\left(h\right)]\approx h^{\wedge }(2p+$2) B + 1/nh V. B and V represent bias and variance, respectively.

Finally, to ensure the consistency of the FRD estimates, the “continuity hypothesis ” needs to be satisfied (56). In this paper, individuals are required not to manipulate the forcing variable, i.e., age, which makes them on the left or right side of the cutoff. Therefore, we use the following two methods to test for continuity: (1) test the continuity of each control variable at the cutoff. If the estimate of the FRD is valid, control variables that are not affected by the NRPS should be continuous at the cutoff. (2) test the continuity of the age density function, i.e., whether age is manipulated. Furthermore, we use a series of robustness tests such as estimation with different bandwidths to ensure the credibility of the results of the FRD.

#### 3.3.2. Benchmark model

Based on the above, we construct the following model to examine the causal effects of the pension from the NRPS on the intake of junk food.


(6) $$\begin{array}{l} Y_{i}\;=\;\alpha +\beta D_{i}+f\left(age_{i}\right)+\gamma Z_{i}+{\omega +\tau +\varepsilon }_{i} \end{array}$$

Where *D*_*i*_ equals 1 if pensions are received and 0 if otherwise. *age*_*i*_ is a forcing variable which represents the respondents' age. *f*(*age*_*i*_) is a polynomial function of *age*_*i*_. *Z*_*i*_ are covariates including gender, education, self-rated health, work status, and household incomes per capita. ω and τ represent province and county fixed effects, respectively. ε_*i*_ is error term.

FRD estimation can be achieved by two-stage least squares (2SLS), which is equivalent to IV estimation (57, 58). Specifically, the one-stage equation can be expressed as follows.


(7) $$\begin{array}{l} D_{i}\;=\;\delta +f\left(age_{i}\right)+\theta T_{i}+\mu_{i} \end{array}$$

Where *T*_*i*_ equals 1 if *age*_*i*_ ≥ 60.25, which is an instrumental variable of *D*_*i*_. μ_*i*_ is error term. The two-stage regression is set up in the same way as equation (6).

## 4. Empirical results

### 4.1. The effect of the age on the intake of junk food

First, we use Logit regression^6^ to examine the effect of the age on the intake of junk food and the results are shown in Table 3. Column (1–4) shows the result of Logit regression when the bandwidths are 15, 8, 6.934, and 5 years, respectively. In Column (1), the OR of the variable *Age_60.25* is 0.859 and significant at the 0.01 level, which indicates that the probability of the intake of junk food in the past week for the samples on the right side of the cutoff is significantly lower than that for the samples on the left side of the cutoff. In addition, the results remain significant at the 0.01 level in Column (2–4) when regression analyses are conducted using subsamples of 8, 6.934, and 5 years on each side of the cutoff. Given that control variables do not affect the consistency of the estimates in the FRD analysis, which are not the focus of this paper, the results of them are not elaborated.

**Table 3:** The effect of the age on the intake of junk food.

**Variables**	**15 years**	**8 years**	**6.934 years**	**5 years**
	**(1)**	**(2)**	**(3)**	**(4)**
Age_60.25	0.859^***^ (0.045)	0.822^***^ (0.058)	0.804^***^ (0.060)	0.744^***^ (0.062)
Gender	1.062 (0.052)	1.058 (0.075)	1.040 (0.080)	1.071 (0.093)
**Education**				
Primary school	0.833^***^ (0.050)	0.858^*^ (0.079)	0.937 (0.092)	0.946 (0.107)
Junior high school	0.836^***^ (0.055)	0.960 (0.095)	0.998 (0.107)	0.969 (0.118)
Senior high school or technical secondary school	0.839^*^ (0.082)	0.871 (0.113)	0.862 (0.120)	0.887 (0.146)
Junior college and above	1.191 (0.538)	0.607 (0.452)	0.591 (0.563)	0.685 (0.684)
**Self-rated health**				
Very healthy	1.016 (0.093)	0.914 (0.129)	0.784 (0.118)	0.789 (0.134)
Healthy	0.955 (0.078)	0.854 (0.109)	0.806 (0.109)	0.781 (0.119)
A little unhealthy	1.084 (0.099)	0.956 (0.133)	0.897 (0.134)	0.926 (0.156)
Unhealthy	0.838^**^ (0.072)	0.799^*^ (0.103)	0.728^**^ (0.100)	0.710^**^ (0.109)
Work status	1.109^*^ (0.070)	1.166 (0.116)	1.209^*^ (0.131)	1.234^*^ (0.154)
Household incomes per capita	1.100^***^ (0.023)	1.108^***^ (0.033)	1.100^***^ (0.035)	1.072^*^ (0.039)
Province fixed effect	Yes	Yes	Yes	Yes
County fixed effect	Yes	Yes	Yes	Yes
Observation	9,927	5,061	4,450	3,473

The values above parentheses represent odds ratio (OR) value which means the relative probability of a variable between a group and a reference group. The reference group for Age_60.25 is those age < 60.25. The reference group for Gender is those who are female, i.e., Gender = 0. The reference group for Education is those who are illiterates, i.e., Education = 1. The reference group for Self-rated health is those who are rather healthy, i.e., Self-rated = 1. The reference group for Work status is those without jobs, i.e., Work status = 0. Analysis in column (1) includes samples aged 45–75 years old, i.e., the bandwidth is 15 years. Analysis in column (2) includes samples aged 52–68 years old, i.e., the bandwidth is 8 years. Analysis in column (3) includes samples aged 53.066–66.934 years old, i.e., the bandwidth is 6.934 years, the minimum mean square error bandwidth. Analysis in column (4) includes samples aged 55–65 years old, i.e., the bandwidth is 5 years. Robust standard errors are clustered at the household level and are shown in parentheses. ^*^p < 0.1, ^**^p < 0.05, ^***^p < 0.01.

Source: CFPS201 and Stata 16.0.

### 4.2. The effect of the NRPS on the intake of junk food

**Figure 2:**
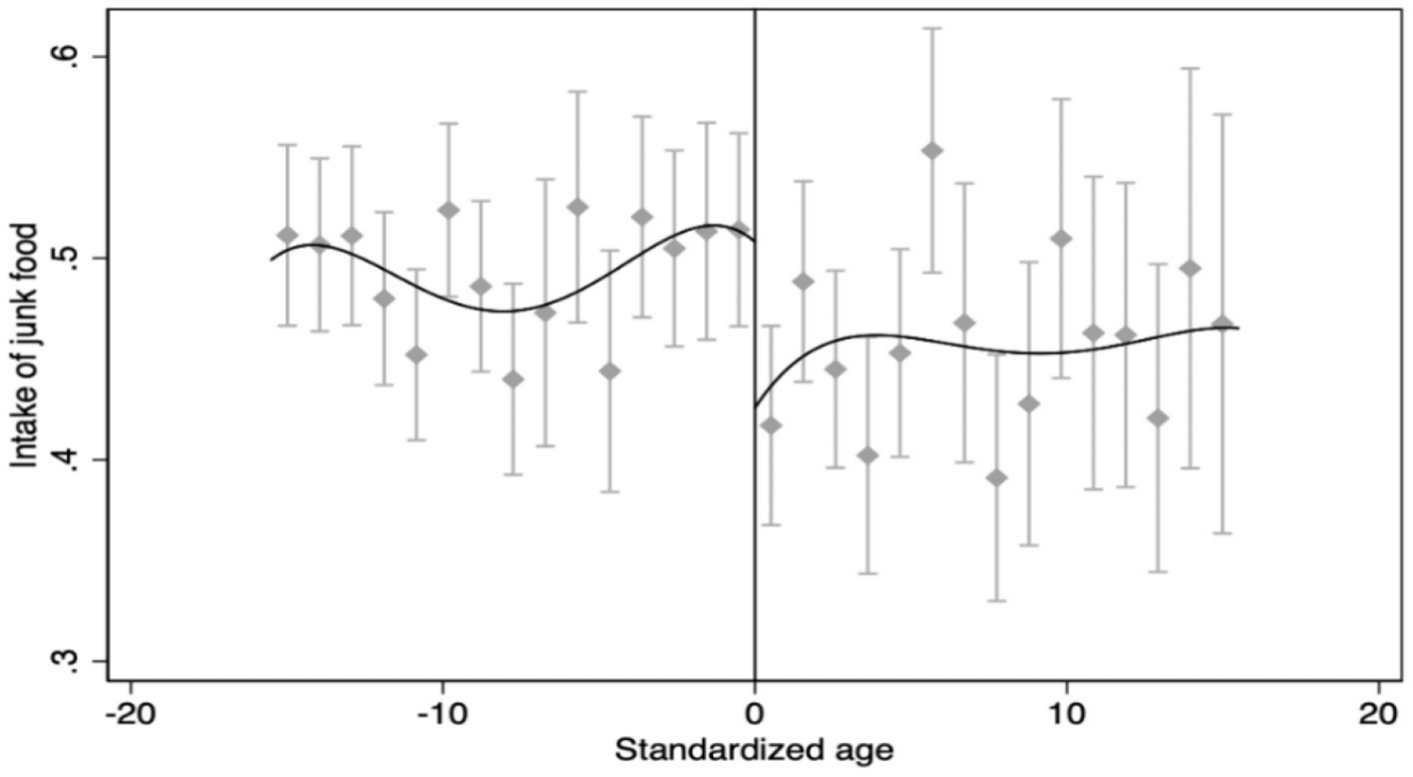
The results of the FRD. Source: CFPS2014 and Stata 16.0.

Then, the results of the FRD are illustrated in Figure 2, showing that the intake of junk food has a clear downward jump at the standardized age 0 point. In the previous section, we have shown the results of the first stage estimation in Figure 1. It suggests that the probability of receiving a pension has a significant upward jump at the standardized age 0 point. Therefore, a preliminary indication can be provided by the two figures that the positive income shock from the NRPS significantly reduces the intake of junk food among rural residents.

Finally, we present the results of FRD regression in Table 4. Column (1) reports the results of the first stage regression. We find that the age specified in the NRPS can significantly increase the probability of receiving pensions by 28.4 percentage points at 0.01 level, which indicates that the cutoff of age can be used as an instrumental variable (IV) for pension receipt. Using the minimum mean square error and calculating the optimal bandwidth (55), we report the result of the benchmark model in Column (2). It shows that the LATE of the FRD is −0.289 and significant at 0.01 level, which suggests that the income from the NRPS results in a significant reduction of the intake of junk food by 0.289.

**Table 4:** The results of FRD regression.

**Variables**	**(1)**	**(2)**
	**Pension**	**Junk food intake**
Age_60.25	0.284^***^ (0.023)	
Pension		−0.289^***^ (0.110)
Covariates	–	Yes
Province fixed effect	–	Yes
County fixed effect	–	Yes
Observation	9,927	2,281; 2,226

The optimal bandwidth is 6.934 years, containing 2,281 and 2,226 samples on the left and right side of the cutoff, respectively. Robust standard errors are clustered at the household level and are shown in parentheses. ^***^p < 0.01.

Source: CFPS2014 and Stata 16.0.

### 4.3. Robust checks

#### 4.3.1. Continuity checks

As mentioned previously, the continuity hypothesis needs to be satisfied for the results of the FRD to be valid. Table 5 reports the results of the continuity check of each covariate at the cutoff. In Column (1–5), the coefficients of covariates are insignificant, which indicates that there is no discontinuity at the cutoff for covariates.

**Table 5:** The results of the continuity check of each covariate at the cutoff.

**Variables**	**(1)**	**(2)**	**(3)**	**(4)**	**(5)**
	**Gender**	**Education**	**Self-rated health**	**Work status**	**Household incomes per capita**
Pension	−0.029 (0.172)	−0.127 (0.276)	−0.658 (0.425)	−0.006 (0.080)	0.578 (0.426)
Bandwidth	4.173	4.994	4.772	6.304	4.231
Covariates	Yes	Yes	Yes	Yes	Yes
Province fixed effect	Yes	Yes	Yes	Yes	Yes
County fixed effect	Yes	Yes	Yes	Yes	Yes
Observation	1,581; 1,477	1,792; 1,734	1,745; 1,664	2,137; 2,083	1,581; 1,477

The default bandwidths of minimum mean square error are used in the table. Robust standard errors are clustered at the household level and are shown in parentheses.

Source: CFPS2014 and Stata 16.0.

**Figure 3:**
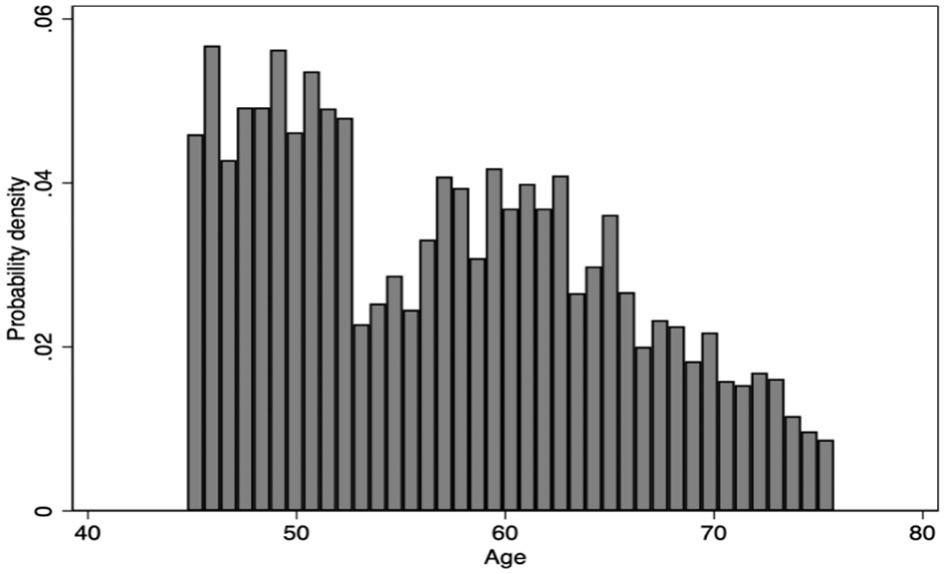
The probability density around age 60. Source: CFPS2014 and Stata 16.0.

Then, we observe whether there is a discontinuity at the cutoff through age probability density plots. The result shown in Figure 3 suggests that the age around the cutoff, i.e., 60 years old, presents roughly an average distribution, which proves that the age is not manipulated. Therefore, the above two testing results have satisfied the premise of continuity hypothesis, which proves that the results of the FRD are valid in this paper.

#### 4.3.2. Regression tests with different bandwidths and orders

In addition to the optimal bandwidth used in the benchmark model regarding the symmetry of both sides of the cutoff, we further exploit the asymmetric optimal bandwidth on both sides (55) as well as the custom bandwidth to re-estimate the benchmark model. The results are shown in Column (1–3) of Table 6. Specifically, Column (1) shows that the LATE of the cutoff is −0.635 and significant at 0.05 level when the optimal bandwidths on the left and right sides are 5.040 and 2.635, respectively. In Column (2) and (3), the coefficients remain negative and significant as the bandwidth is set as 5 and 8 years, respectively. Besides, as shown in columns (4–6) of Table 6, we further set the regression order as 2 and use the asymmetric optimal on both sides of the cutoff as well as the custom bandwidth to re-estimate our benchmark model. The coefficients are roughly negative and significant. In summary, the above results with different bandwidths and orders imply that the positive income shock from the NRPS can significantly reduce the intake of junk food, which further demonstrates the robustness of the benchmark.

**Table 6:** Regression tests with different bandwidths and orders.

**Variables**	**(1)**	**(2)**	**(3)**	**(4)**	**(5)**	**(6)**
Pension	−0.635^**^ (0.289)	−0.321^**^ (0.156)	−0.272^***^ (0.095)	−0.751^*^ (0.413)	−0.758^#^ (0.488)	−0.433^*^ (0.241)
Order	1	1	1	2	2	2
Bandwidth	5.040; 2.635	5	8	6.414; 5.112	5	8
Covariates	Yes	Yes	Yes	Yes	Yes	Yes
Province fixed effect	Yes	Yes	Yes	Yes	Yes	Yes
County fixed effect	Yes	Yes	Yes	Yes	Yes	Yes
Observation	1,809; 1,018	1,792; 1,734	2,602; 2,454	2,169; 1,787	1,792; 1,734	2,602; 2,454

The default bandwidths of minimum mean square error are used in the table. Covariates include gender, education, self-rated health, work status, and household incomes per capita. Robust standard errors are clustered at the household level and are shown in parentheses. ^#^*p* < 0. 12, ^*^p < 0.1, ^**^p < 0.05, ^***^p < 0.01.

Source: CFPS2014 and Stata 16.0.

#### 4.3.3. Placebo tests

In this section, we perform placebo tests by setting pseudo-cutoffs. The rationale is that if the coefficients are not significant at any of pseudo-cutoffs, it is indeed the positive income shock from the NRPS instead of other factors that influences the intake of junk food. Therefore, we set setting pseudo-cutoffs at ages 56, 57, 58, 62, 63, and 64, respectively, and re-estimate the benchmark model. The results in Table 7 show that the coefficients are not significant at any of pseudo-cutoffs we set, which indicates that the reduction of junk food after 60 years old is indeed due to the NRPS.

**Table 7:** Placebo tests.

**Variables**	**56**	**57**	**58**	**62**	**63**	**64**
	**(1)**	**(2)**	**(3)**	**(4)**	**(5)**	**(7)**
Pension	7.298 (10.022)	−4.601 (7.557)	0.598 (0.920)	0.411 (0.285)	−19.133 (1,915.200)	0.481 (1.194)
Bandwidth	2.391	2.474	3.541	2.430	2.080	3.042
Covariates	Yes	Yes	Yes	Yes	Yes	Yes
Province fixed effect	Yes	Yes	Yes	Yes	Yes	Yes
County fixed effect	Yes	Yes	Yes	Yes	Yes	Yes
Observation	627; 898	707; 929	1,140; 1,355	938; 834	784; 612	1,064; 849

The default bandwidths of minimum mean square error are used in the table. Covariates include gender, education, self-rated health, work status, and household incomes per capita. Robust standard errors are clustered at the household level and are shown in parentheses.

Source: CFPS2014 and Stata 16.0.

#### 4.3.4. Further discussion of the plausibility of the results

In this section, we provide our explanations for three potential questions that may arise from the results of this paper. Firstly, participants of the NRPS are aware of the regulations on when the pensions can be received, which thus may cause them to change their behavior before receiving their pensions. If so, theoretically, we will expect such behavior to have a positive effect on the intake of junk food. However, the coefficient of the FRD is significantly negative. Therefore, we consider that this result should only be a lower bound on the NRPS, which means that the actual effects should be much stronger. Secondly, are there other policies in rural China that are implemented at the age 60 cutoff, which thus have an impact on the NRPS? According to Chen (59), there are no other policies implemented in rural China for the older adults at age 60. Therefore, it can be proved that, we think, the above results are indeed brought by the NRPS. Thirdly, does the NRPS really have an income shock? In fact, some previous studies have shown that the NRPS does significantly increase the income level of participants (26, 60), resulting in an income shock. In summary, we believe that our conclusion is robust and the NRPS does significantly reduce the intake of junk food among rural residents in China.

## 5. Heterogeneous analysis

Considering that the effects of the pension shock by the NRPS on the intake of junk food may be heterogeneous due to different individual characteristics. Therefore, we respectively divide the total sample into subsamples according to gender, education, work status, and household incomes per capita, which can enrich the findings in this paper. The results of the heterogeneity are reported in Table 8 and the heterogeneous analysis is as follows.

**Table 8:** Heterogeneous analysis.

**Variables**	**Pension**	**Bandwidth**	**Covariates**	**Province fixed effects**	**County fixed effects**	**Observation**
**Gender**						
Male	−0.245^*^ (0.142)	6.488	Yes	Yes	Yes	1,102; 1,067
Female	−0.368^*^ (0.205)	6.280	Yes	Yes	Yes	1,055; 1,028
**Education**						
Junior high school and below	−0.286^**^ (0.136)	5.863	Yes	Yes	Yes	1,754; 1,957
Senior high school and above	−1.827 (2.256)	3.716	Yes	Yes	Yes	149; 41
**Work status**						
Jobs	−0.436^**^ (0.206)	4.236	Yes	Yes	Yes	1,306; 1,291
No jobs	0.045 (0.609)	4.688	Yes	Yes	Yes	300; 199
**Household incomes per capita**						
Low-income	−0.326^*^ (0.174)	6.347	Yes	Yes	Yes	712; 787
Middle-income	−0.403 (0.720)	3.368	Yes	Yes	Yes	458; 410
High-income	−0.345 (0.274)	5.574	Yes	Yes	Yes	634; 556

The default bandwidths of minimum mean square error are used in the table. Covariates include gender, education, self-rated health, work status, and household incomes per capita. Robust standard errors are clustered at the household level and are shown in parentheses. ^*^p < 0.1, ^**^p < 0.05.

Source: CFPS2014 and Stata 16.0.

### 5.1. Heterogeneity in gender

From the perspective of gender, the intake of junk food is significantly affected by the NRPS for both males and females. Specifically, the LATE for males and females is respectively −0.245 and −0.368, and both are significant at 0.1 level, which suggests that females are more responsive to the income shock of the NRPS on the intake of junk food.

### 5.2. Heterogeneity in education

From the perspective of education, the LATE for those who receive junior high school and below education is −0.286 and significantly at 0.05 level. By contrast, the LATE for those who receive senior high school and above education is not significant. The above results, we think, are in line with our expectations. The reason is that education is generally proportional to income and socioeconomic status. Thus, the lower the education level, the more sensitive it is to the income shock from the NRPS.

### 5.3. Heterogeneity in work status

From the perspective of work status, the LATE for those without jobs is −0.436 and significantly at 0.05 level. By contrast, the LATE for those who have jobs is not significant. The above results are consistent with our expectations as well. Compared with those who have jobs, those without jobs represent lower income and socioeconomic status to some extent, which leads to them more responsive to the income shock of the NRPS on the intake of junk food.

### 5.4. Heterogeneity in household incomes per capita

Income is a significant factor influencing people's consumption of food (61). Generally, households with lower income are more responsive to income shocks, and vice versa (28). To verify this, we divide the total samples into low-, middle-, and high-income groups according to their household incomes per capita by means of quartile. The LATE for the low-income group is −0.436 and significantly at 0.1 level. By contrast, the LATE for the middle- and high-income groups is not significant. Therefore, low-income households are more responsive to the income shock of the NRPS on the intake of junk food, which further confirm our expectations.

## 6. Discussion

With the rapid development of economy and society, people pay more attention to their health, and dietary health is an important part of it. Previous studies have already told us that the improvement of dietary health and nutritional structure not only contributes to sustainable socio-economic development at the macro level (62), but also contributes to the increase of human capital at the micro level (63). Therefore, how to promote dietary health from different aspects has become a key research topic for many scholars. In this section, we will compare the findings of this paper to the existing literature and elaborate on the possible limitations of this paper and the prospects for future study.

### 6.1. Comparison to the existing literature

To our knowledge, this is the first study to evaluate the effects of the NRPS from the perspective of dietary health. Our FRD results show that the pension shock from the NRPS can significantly reduce the intake of junk food among rural older adults in China. A great number of studies have shown that reducing the intake of junk food can be effective in improving health outcomes (11, 12). Our results are in line with findings for the NRPS showing that the NRPS improves the nutrition intake and health status (26, 41, 43).

Considering the negative correlation between junk food intake and health status, our results are consistent with the role of the pension in health status improvement in different countries. For instance, the social pension in Korea may have benefitted the health of the beneficiaries by improving their nutrition (27). The social pensions in South Africa improve health of older persons (64). Our results provide a potential mechanism and interpretation for them, i.e., improving health by reducing unhealthy food.

Our heterogeneity analysis shows heterogeneous effects of the NRPS on the intake of junk food in terms of gender, education level, work status, and income. Firstly, with regards to gender, our result shows that the effects of the NRPS on females are more pronounced, which is in line with the relevant findings. There are distinct gender differences in dietary patterns in China (65), and females' dietary status are overall poorer than males' (35). In addition, rural females are likely to consume low-nutrition foods due to cognitive limitations and less exposure to knowledge related to healthy and balanced diets, which thus makes them more sensitive to the pension from the NRPS. Secondly, with regards to education level, our result shows that the low-educated are more sensitive to the pension from the NRPS. The possible reason is that education level is related to consumption perception and purchasing power. Specifically, the high-educated attach importance to health and nutrition and tend to purchase healthier foods at higher prices as a result of their higher level of self-health awareness and increased nutrition knowledge (66, 67). By contrast, those with less education are apt to consume unhealthy foods. Thirdly, with regards to work status and income, it is unsurprising that the effects of the NRPS are more significant for the unemployed and low-income. Work is the way to obtain income. Existing studies have shown that income is one of the main factors influencing dietary quality (66, 68–70). Low-income older adults have poor nutrient intake and dietary quality (71). Therefore, work and improved income will undoubtedly make people underline the nutrition of food and their own health, which contributes to better dietary quality. Instead, the unemployed and low-income are likely to consume unhealthy food (72), which thus makes them more responsive to the pension from the NRPS.

### 6.2. Limitations and further studies

Nevertheless, this study has three possible limitations. Firstly, the effects of the NRPS on the past 7 days' diets could only be examined, so we have no way to verify the long-term effects and the mechanism due to data limitations. Secondly, some confounding effects caused by unobservable time-varying factors are possibly inevitable with cross-section data in this paper, though FRD helps to control observable and unobservable factors. Thirdly, the heterogeneity analysis of this study shows that the pension shock from the NRPS is stronger for the relatively vulnerable groups, which thus leads to the results unable to be generalized to the whole population.

Therefore, we expect that future studies will use more comprehensive data to verify the long-term effects and mechanisms of the NRPS. In addition, studies with different perspectives are needed if we are to understand how to improve the dietary quality for the whole population.

## 7. Conclusion

Given that there were few studies examining that effects of the pension scheme on diary health, using the pension shock from China's NRPS, we conduct a FRD to address possible endogeneity and examine the causal effect of the NRPS on junk food intake among rural residents by using the data China Family Panel Studies (CFPS) from 2014.

We have two main findings. First, FRD shows that pensions from the NRPS can effectively reduce the intake of junk food among the rural older adults in China. The result remains robust after a series of robustness checks. Therefore, we have reason to believe that increasing the income level of the rural older adults can effectively improve their dietary health. Second, there is heterogeneity in the effects of the NRPS on the intake of junk food. Meanwhile, the results indicate that there is an unreasonable gap in dietary health among different groups of the rural older adults in China as well. Specifically, the mitigating effects is stronger for female, low-educated, unemployed, and low-income groups, which are consistent with the intuition that relatively vulnerable groups tend to more influenced by the NRPS.

We believe these findings can be of interest to policymakers and provide them with some policy insights. Specifically, first, government should endeavor to increase the level of pension benefits to achieve a better improvement of dietary health among the rural older adults. Second, government should take some reasonable measures to reduce dietary health inequalities. For instance, the enrollment rate of the female, low-educated, unemployed, and low-income groups should be improved, which requires more attention from the relevant authorities. In addition, encourage seniors to receive education from senior colleges. Besides, more jobs should be created to achieve re-employment of older adults. Finally, appropriately increase government assistance and subsidies to older adults who are extremely poor.

## Author contributions

SP: methodology, validation, resources, and funding acquisition. ZS, JC, and ZL: writing—original draft preparation. ZS: writing—review and editing. ZL: software. All authors have read and agreed to the published version of the manuscript.

## Footnotes

^1^The UEBEI provides pension security for urban employees in enterprises or administrative institutions. It is also a vital part of China's pension system.

^2^The reason for intercepting age 75 on the right-hand side of the cutoff at age 75 is that age 75 is symmetric with respect to the cutoff at age 45. Since the FRD is a weighted “local” treatment effect, ultimately only the local sample within the bandwidth is causally inferred. Moreover, the results will be robustly tested for different bandwidths in Section 4.3.2. Therefore, the interception of sample age does not affect the conclusions of this paper. If other age is intercepted on the right-hand side of the cutoff, the results are basically identical.

^3^Another reference method in nutrition studies is called 24-hour recall method which is applied to studies in nutrition as well (e.g., 50, 51). It is a method to calculate the food intake by inquiring their actual dietary intake of the respondents in the past 24 hours. Due to questionnaire limitations, the 2014 CFPS does not provide information on 24-hour dietary intake, which is why we could only use the 7-day dietary intake in this study.

^4^In fact, FRD has been widely used to analyze pension schemes in China, such as (38, 41, 43, 52).

^5^If the breakpoint is set to 60.5 or 60.3, the result remains unchanged.

^6^Due to the limitation of length and the fact that this method is not the focus of this paper, the result of Logit only serves as a pre-test and is therefore not mentioned in the empirical strategy. In this section, our purpose is to initially observe the difference between the left and right sides of the cutoff, which can also be interpreted as a preliminary test of the effect of NRPS on the intake of junk food using age as a proxy.
